# Use of a Hydrophobic Azo Dye for the Centrifuge-Less Cloud Point Extraction–Spectrophotometric Determination of Cobalt

**DOI:** 10.3390/molecules27154725

**Published:** 2022-07-24

**Authors:** Kiril Blazhev Gavazov, Petya V. Racheva, Nikolina P. Milcheva, Vidka V. Divarova, Denitsa Dimitrova Kiradzhiyska, Fatma Genç, Antoaneta D. Saravanska

**Affiliations:** 1 Department of Chemical Sciences, Faculty of Pharmacy, Medical University of Plovdiv, 120 Buxton Bros Str., 4004 Plovdiv, Bulgaria; petya.racheva@mu-plovdiv.bg (P.V.R.); vidka.divarova@mu-plovdiv.bg (V.V.D.); denitsa.kiradzhiyska@mu-plovdiv.bg (D.D.K.); antoaneta.saravanska@mu-plovdiv.bg (A.D.S.); 2 Faculty of Pharmacy, İstanbul Yeni Yüzyıl Üniversitesi, 26 Yılanlı Ayazma Caddesi, 34010 İstanbul, Turkey; ftmgenc@yahoo.com

**Keywords:** cobalt, cloud point extraction, green analytical chemistry, azo dye, spectrophotometric determination

## Abstract

The hydrophobic azo dye 6-hexyl-4-(2-thiazolylazo)resorcinol (HTAR, H_2_L) was studied as part of a system for the centrifuge-less cloud point extraction (CL-CPE) and spectrophotometric determination of traces of cobalt. The extracted 1:2 (Co:HTAR) complex, [Co^III^(HL^−^)(L^2−^)]^0^, shows an absorption maximum at 553 nm and contains HTAR in two different acid–base forms. Optimum conditions for its formation and CL-CPE were found as follows: 1 × 10^−5^ mol L^−1^ of HTAR, 1.64% of Triton X-114, pH of 7.8, incubation time of 20 min at ca. 50 °C, and cooling time of 30 min at ca. −20 °C. The linear range, limit of detection, and apparent molar absorptivity coefficient were 5.4–189 ng mL^−1^, 1.64 ng mL^−1^, and 2.63 × 10^5^ L mol^−1^ cm^−1^, respectively. The developed procedure does not use any organic solvents and can be described as simple, cheap, sensitive, convenient, and environmentally friendly. It was successfully applied to the analysis of artificial mixtures and real samples, such as steel, dental alloy, rainwater, ampoules of vitamin B_12_, and saline solution for intravenous infusion.

## 1. Introduction

Cobalt is a group 9 first-row transition metal with atomic number 27. It is a hard, lustrous, silvery-gray, corrosion-resistant ferromagnetic material, recognized as a new element (1735) by the Swedish chemist Georg Brandt. Pure metal is not found in nature, but its compounds are part of most rocks and soils [1,2]. It is classified as a dispersed element with an average content in the upper crust of 17.3 ppm [3,4]. Cobalt is the essential ingredient of 72 approved minerals in the International Mineralogical Association’s database [5], the most important of which are cobaltite (Co^III^AsS), glaucodot (Co^III^FeAsS), erythrite (Co^II^_3_(AsO_4_)_2_⋅8H_2_O), and skutterudite (Co^III^As_3_). However, its economically viable deposits are few and cobalt is usually produced as a by-product of other metals (e.g., Ni, Cu, and Ag) [6,7].

The main applications of cobalt are in rechargeable batteries, superalloys, steels, magnets, pigments, chemicals, ceramics, catalysts, oil drying agents (siccatives), and nutrients [7,8,9]. As the need for rechargeable batteries continues to grow, some economists expect a significant increase in demand for this element in the coming years [8,10].

Cobalt is an essential trace element for many organisms, including humans. It is utilized by animals only in the form of vitamin B_12_ (cobalamin), synthesized by specific microorganisms in the presence of sufficient cobalt. This vitamin is important for the development, myelination and function of the central nervous system, DNA synthesis, and red blood cell formation [11]. Cobalt deficiency in humans can lead to health problems, such as pernicious anemia, nerve damage, and reduced resistance to parasite and microbial infections [12,13]. On the other hand, excessive cobalt, which is often the result of human activity, can cause systemic toxicity affecting multiple organ systems [14,15].

Numerous spectroscopy techniques have been used for the determination of cobalt, including flame atomic absorption spectrometry (FAAS) [16,17,18,19], graphite furnace atomic absorption spectrometry (GFAAS) [20], inductively coupled plasma mass spectrometry (ICP-MS) [21], inductively coupled plasma optical emission spectrometry (ICP-OES) [22], and thermal lens spectrometry [23].

UV/Vis spectrophotometry is considered a good alternative for the determination of inorganics, thanks to its simplicity, low cost, versatility, energy efficiency, sensitivity, and availability [24,25,26]. It can be easily combined with various sample preparation techniques to improve analytical characteristics and extend the scope of application. The list of such techniques comprises liquid–liquid extraction [27], dispersive liquid–liquid microextraction [28], continuous sample drop flow-based microextraction [29], deep eutectic solvent microextraction [30], and cloud point extraction (CPE) [31,32,33,34,35,36,37,38,39,40,41,42,43,44].

CPE is a modern variant of the classical liquid–liquid extraction, which minimizes (or completely ignores) the use of organic solvents. It complies with the Green Analytical Chemistry principles [45] and is often defined as an “eco-friendly tool” [46]. The application of CPE to trace element analysis usually requires the conversion of the analyte into a hydrophobic electroneutral complex [47,48,49] that enters the surfactant-rich phase (SRP). This phase is typically separated by centrifugation [49,50], but sometimes a simpler option is possible: separation based on gravitational forces. Given the proper choice of reagent(s) and experimental conditions, gravitational (centrifuge-less, CL) separation is convenient and advantageous, and the time required for the process to complete is not very long.

**Figure 1:**
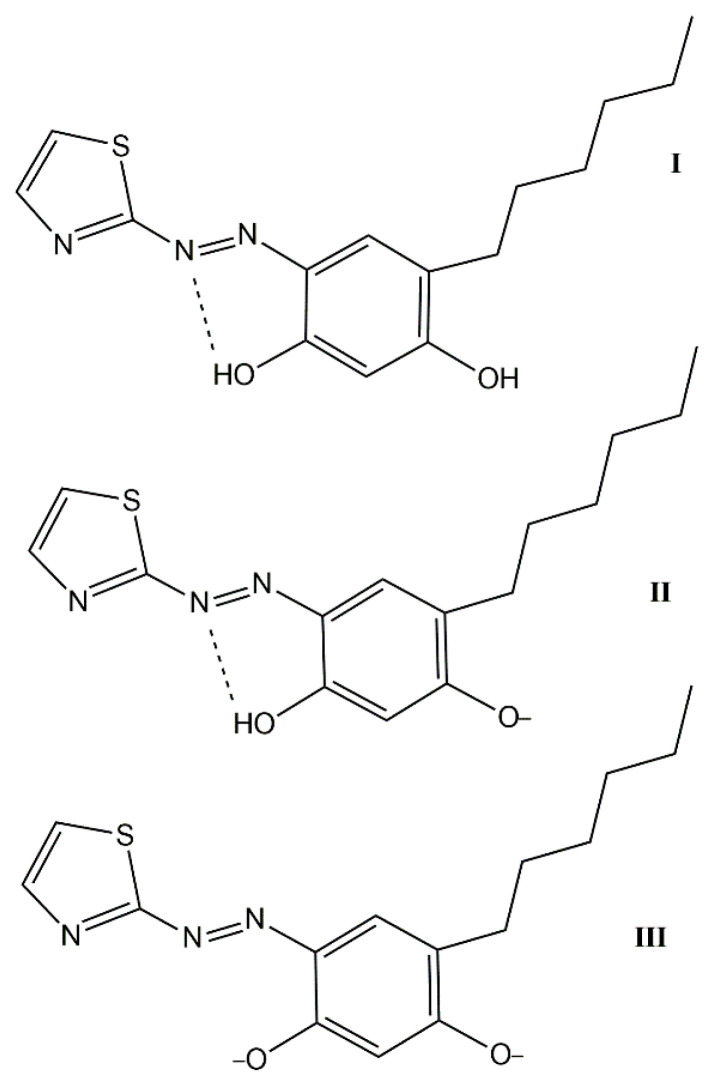
Chemical formulae of the HTAR forms: neutral, H_2_L (**I**); monoanionic, HL^−^ (**II**); and dianionic, L^2^^−^ (**III**).

The aim of this work was to develop a CL-CPE–spectrophotometric procedure for the determination of traces of cobalt, using 6-hexyl-4-(2-thiazolylazo)-resorcinol (HTAR). This novel hydrophobic reagent (Figure 1) has recently been applied in our laboratory for the CL-CPE of vanadium(IV,V) [51] and copper(II) [52].

## 2. Results and Discussion

### 2.1. Optimimum Conditions

A single factor optimization was carried out in this study. The influence of the following experimental parameters was examined and optimized at room temperature: wavelength of spectrophotometric measurement, pH, HTAR concentration, and TX-114 mass fraction. The effect of incubation time at elevated temperature (50 °C) was also investigated.

**Figure 2:**
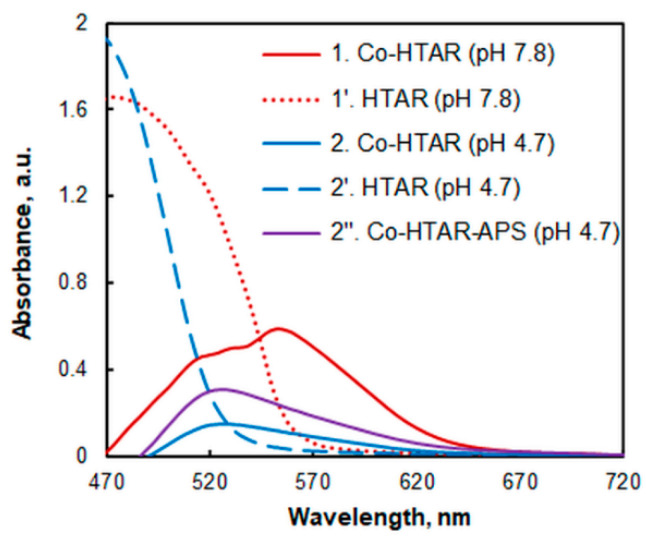
Absorption spectra of the complex (1, 2, 2”) and the blank (1’, 2’) at two different pH values (ammonium acetate buffer): 7.8 (1, 1’) and 4.7 (2, 2’, 2”). 2.25 × 10^−6^ mol L^−1^ of Co(II), 1 × 10^−5^ mol L^−1^ of HTAR, 1.64% of TX-114, *t* = 20 min at 50 °C. The concentration of APS (2”) was 1.6 × 10^−4^ mol L^−1^.

Spectra of the extracted species are shown in Figure 2. The Co–HTAR coordination compound obtained in neutral or basic media shows an absorption maximum (*λ*_max_) at 553 nm (1). Spectral characteristics do not change when a strong oxidizing agent (e.g., ammonium persulfate, APS) is added to the system (the resulting spectral line is identical to spectrum 1). This suggests that the central atom is Co(III), a statement consistent with the literature describing a spontaneous Co(II) → Co(III) oxidation reaction with the dissolved oxygen in systems containing azo dyes [53,54,55,56,57,58].

In acidic solutions, the recorded spectrum of the Co–HTAR complex (2) changes to *λ*_max_ = 524 nm. This hypsochromic shift can be attributed to the lower absorbance of the blank (2’), which is determined by the existence of the HTAR reagent mainly in its neutral form (H_2_L) at pH < 6.7 [52]. The addition of APS does not affect the position of λ_max_, but the absorbance becomes higher (compare spectra 2 (Co-HTAR) and 2” (Co-HTAR-APS)). This is consistent with the fact noted by many authors that the oxidation of Co(II) is not as fast and easy at lower pH values.

**Figure 3:**
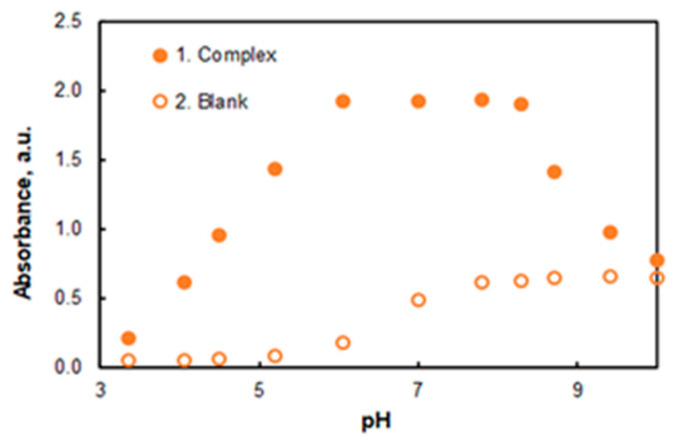
Effect of pH on the absorbance: 8 × 10^−6^ mol L^−1^ of Co, 2.8 × 10^−5^ mol L^−1^ of HTAR, 2.0% of TX-114, *t* = 20 min at 50 °C, *λ* = 553 nm.

The effect of pH on the absorbance at 553 nm is shown in Figure 3. An ammonium acetate buffer (3 mL) was used to adjust the pH. The absorbance is maximal and constant in a wide pH range (from 6.0 to 8.3). Further studies were performed at pH 7.8. This pH value was chosen based on the following considerations: (a) it is far from the limit values (6.0 and 8.3); (b) the absorbance of the blank (at *λ* = 553 nm) is weakly sensitive to accidental pH deviations.

**Figure 4:**
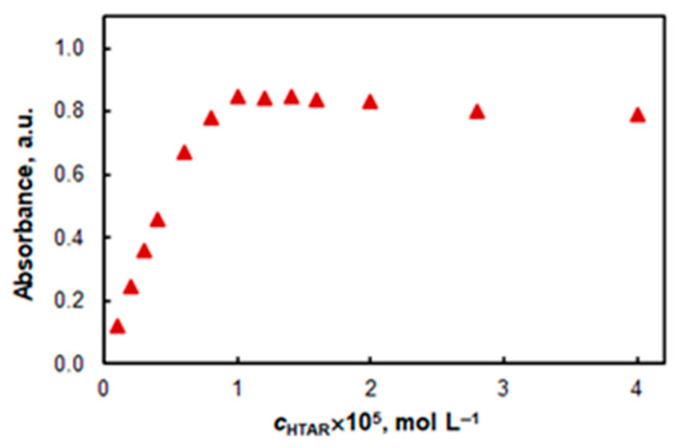
Effect of HTAR concentration: 1.6% of TX-114, 3.2 × 10^−6^ mol L^−1^ of Co, pH 7.8, *t* = 20 min at 50 °C, *λ* = 553 nm.

The effect of HTAR concentration is displayed in Figure 4. The chosen optimal concentration was 1.0 × 10^−5^ mol L^−1^. At concentrations above (1.5–2.0) × 10^−5^ mol L^−1^, a slight decrease in absorption is observed.

**Figure 5:**
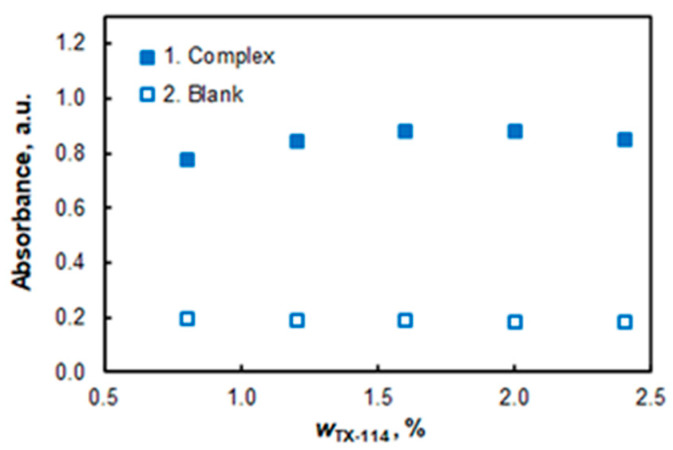
Effect of TX-114 mass fraction: 3.2 × 10^−6^ mol L^−1^ of Co, 1 × 10^−5^ mol L^−1^ of HTAR, pH 7.7, *t* = 20 min at 50 °C, *λ* = 553 nm.

The effect of the Triton X-114 (TX-114) mass fraction is demonstrated in Figure 5. Further studies were performed in the presence of 8.2 mL (≈8.2 g) of the surfactant solution, which corresponds to an approximate mass fraction of 1.64%.

**Figure 6:**
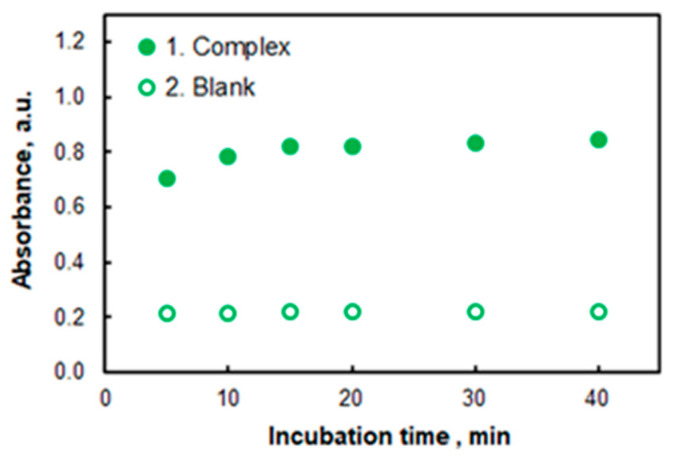
Effect of incubation time (t) at 50 °C: 1.6% of TX-114, 3.2 × 10^−6^ mol L^−1^ of Co, pH 7.7, 1 × 10^−5^ mol L^−1^ of HTAR, *λ* = 553 nm.

The reported heating temperatures for CPE systems based on the same surfactant, TX-114, are commonly between 40 °C and 65 °C [23,31,32,35,36,38,39,59,60,61]. The results of our experiments at 50 °C are represented in Figure 6. As can be seen, the minimum incubation time required is ca. 15 min. To avoid accidental errors caused by insufficient heating, further studies were performed at an incubation time of 20 min.

The last step of the developed CL-CPE procedure is cooling. The samples were kept in a refrigerator (at ca. −20 °C) for 30 min, as in our previous work on Cu(II) extraction with the same surfactant (TX-114) and reagent (HTAR) [52]. 

The selected optimal CL-CPE–spectrophotometric conditions are summarized in Table 1.

### 2.2. Composition of the Complex, Formula, Extraction Equation, and Equilibrium Constant

**Figure 7:**
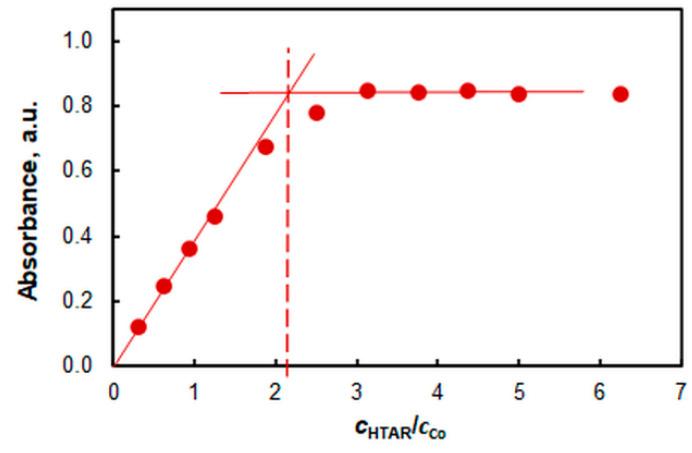
Determination of the HTAR:Co molar ratio by the mole-ratio method: 1.6% of TX-114, 3.2 × 10^−6^ mol L^−1^ of Co, pH 7.8, *t* = 20 min at 50 °C, *λ* = 553 nm.

**Figure 8:**
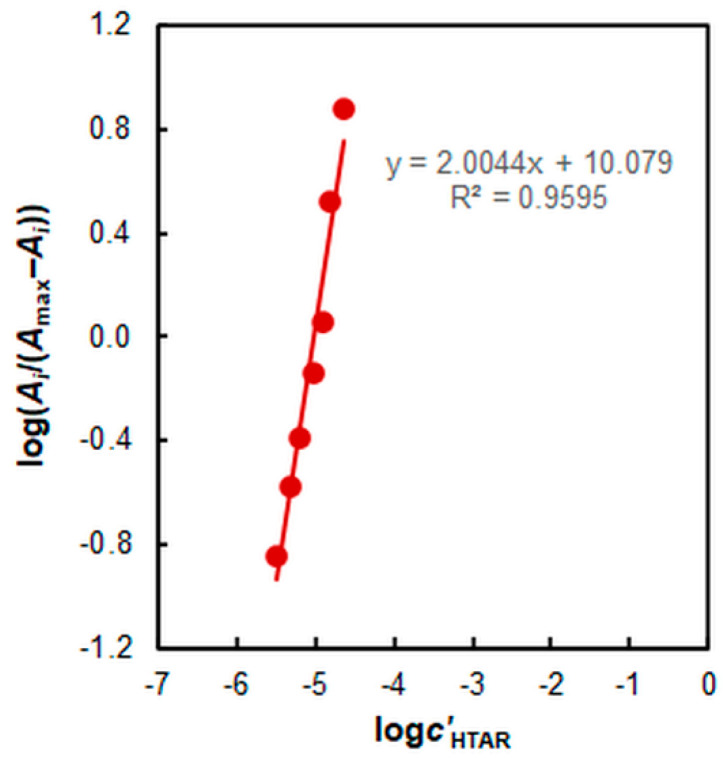
Determination of the HTAR:Co molar ratio by the mobile equilibrium method: 1.6% of TX-114, 3.2 × 10^−6^ mol L^−1^ of Co, pH 4.7, *t* = 20 min at 50 °C, *λ* = 553 nm.

The complex stoichiometry was determined by the mole-ratio method [62] and the mobile equilibrium method [63] at two different pH values (7.8 and 4.7). A molar ratio of 1:2 (Co:HTAR) was found regardless of pH (Figure 7 and Figure 8). 

Based on the electroneutrality requirement and the lack of indications that the components of the buffer are included in the complex, one can suggest the following formula of the extracted coordination compound: [Co^III^(HL^–^)(L^2–^)]^0^. Complexes with such a general formula, containing one deprotonated (L^2–^) and one monoprotonated (HL^–^) azo dye, have been partially extracted in water–chloroform systems involving similar thiazolylazo dyes, such as 4-(2-thiazolylazo)resorcinol (TAR) [55] and 5-methyl-4-(2-thiazolylazo)resorcinol (MTAR) [58].

The extraction process in the studied system (at the optimum pH range, 6.0–8.3) can be expressed by the following equation, involving oxidation of Co(II) to Co(III):

Co^II^_(aq)_ + 2 HL^−^_(aq)_ → [Co^III^(HL^−^)(L^2−^)]_(SRP)_ + H^+^_(aq)_ + e^−^

At lower pH values, the complexation is hampered by HL^–^ deficiency (H_2_L is the dominant species) and incomplete Co(II) oxidation. At higher pH values, the main obstacles may be hydrolysis [64] and HL^−^ deficiency (due to an increase in the L^2−^ fraction).

The equilibrium constant characterizing this equation was calculated by the Harvey–Manning method [65], log*K*_ex_ = 12.1 ± 0.2 (mean ± standard deviation).

### 2.3. Analytical Characteristics, Effect of Foreign Ions and Application

The relationship between the absorbance and concentration of Co(II) was investigated under the optimal conditions given in Table 1. Good linearity was found in the range of 5.4–189 ng mL^−1^, *R*^2^ = 0.9992 (*n* = 8). The regression equation was *A* = 4.460*γ* + 0.0047, where *γ* is the concentration in μg mL^−1^. The standard deviations of the slope and intercept were 0.052 and 0.0049, respectively. The molar absorption coefficient was 2.63 × 10^5^ L mol^−1^ cm^−1^, and the limits of detection (LOD) and quantitation (LOQ), calculated as 3- and 10-times standard deviation of the blank divided by the slope, were 1.64 ng mL^−1^ and 5.4 ng mL^−1^, respectively. The preconcentration factor defined as the ratio between the masses of the sample (50 g ≈ 50 mL; the density is close to unity) and the diluted SRP phase (5 g) was 10.0. A similar value (10.8) was calculated by dividing the slopes obtained in the presence and absence of TX-114.

The effect of foreign ions is shown in Table 2. The most serious interferences are caused by Cu(II), Ni(II), and Zn(II). Under the established optimum conditions, these ions form colored complexes with absorption maxima at 547 nm (Cu), 554 nm (Ni), and 533–543 nm (Zn). The interfering effect of V(V) and Fe(III) is smaller. If necessary, Fe(III) can be masked with HPO_4_^2−^ [66] or separated by the fluoride method [67], as described below.

The developed procedure was used to determine Co in artificial mixtures and real samples. As a first step, artificial mixtures imitating cobalt-based dental and super-alloys were analyzed: Marranium CC, EOS CobaltChrome SP2, Vitallium, and Stellite 6. The results were statistically identical to those obtained with the same amount of Co(II) (4.7 μg) and the absence of ions corresponding to the alloying elements. The relative standard deviation (RSD) was in the range of 1.2–1.7% (*n* = 4).

Table 3 and Table 4 show the results of the analysis of real samples: steel, dental alloy, and injection ampoules of Vitamin B_12_. They characterize the developed procedure as accurate and precise. The results for the cobalt content of the injection ampoules were additionally confirmed by ICP-MS.

Table 5 includes the results of analysis of rainwater and saline solution for intravenous infusion obtained by the addition–recovery method. The RSD in these determinations ranged from 2.4% to 27%, and the recoveries were between 98.2% and 106%.

### 2.4. Comparison with Existing Methods

Table 6 summarizes data on CPE–spectrophotometric procedures for the determination of cobalt. The present CL-CPE procedure can be described as simple, cheap, sensitive, convenient, and environmentally friendly. The reagent is commercially available and does not need to be synthesized. It is not necessary to add electrolyte to increase the extraction efficiency [36,37,40,41] or organic solvent to provide synergistic extraction [33] or to reduce the viscosity of the SRP [31,32,33,34,35,36,37,38,39,40,41].

## 3. Materials and Methods

### 3.1. Chemicals and Instrumentation

The chemicals were purchased from Merck (Germany). The stock Co(II) solution (1000 mL, 1 mg mL^–1^) was prepared by dissolving cobalt(II) sulfate heptahydrate in water containing 2 mL of conc. H_2_SO_4_ [24]. Working 4 × 10^−4^ mol L^−1^ Co(II) solutions were obtained by appropriate dilution with water. An aqueous solution of HTAR (2 × 10^−3^ mol L^−1^) was prepared in the presence of KOH [52]. Laboratory grade TX-114 was used. It was diluted with water at a mass fraction of 10%. Buffer solutions were made by mixing appropriate volumes of aqueous solutions (2 mol L^−1^) of ammonia and acetic acid. Distilled or deionized (ELGA-Veolia LabWater, UK) water was used during the experiments.

An Ultrospec 3300 pro (United Kingdom), equipped with 1 cm path-length cells, was used for the spectrophotometric measurements. The pH was checked with a WTW InoLab 7110 pH meter (Germany). The samples were heated in a GFL 1023 water bath (Germany). An Ohaus Pioneer PA214C analytical balance (USA) was used to measure the mass.

### 3.2. Samples and Sample Preparation

A saline solution of 0.9% NaCl for intravenous infusion (1000 mL) and 1.0-mL ampoules of vitamin B_12_ (solution for injection, 1000 μg vitamin B_12_) were purchased from a local pharmacy. A dental alloy (Wirobond® C) was kindly provided by the Research Institute at Medical University of Plovdiv. A standard steel sample (4.71% Co) was supplied by the KCM S.A.–Plovdiv.

Rainwater (pH ca. 6.0) was sampled in the outskirts of Plovdiv, Bulgaria (12 June 2022) during the Mediterranean cyclone called “Genesis”. A PET bottle and a glass funnel were used during sampling. The analysis was performed the next day using 35-mL aliquots.

The saline solution for intravenous infusion was also analyzed using 35-mL aliquots.

Vitamin B_12_ ampoules were prepared for analysis by the procedure [68] involving heating in a mixture of conc. HNO_3_ (10 mL) and conc. H_2_SO_4_ (1 mL) to dryness on a sand bath. The volume of the final solution was 50 mL, and 2-mL aliquots were taken for the analysis.

The dental alloy was treated as described in Ref. [69]. An accurate amount of the alloy (*ca.* 0.05 g) was weighed into a 50 mL beaker. Then, 10 mL of aqua regia was added and the sample was heated on an initially cold sand bath to dryness. After cooling, 5 mL of HCl (1:1) was added. The sample was reheated to dryness, and the resulting salts were dissolved in water. The obtained solution was transferred to a 1000-mL volumetric flask, and water was added to the mark. Aliquots of 0.2 mL were used for the analysis. 

The steel (*ca.* 0.5 g) was dissolved by a known procedure [70,71,72] and collected in a 1000-mL volumetric flask. The fluoride precipitation method [67] (p. 177) was then used to remove Fe(III). For this purpose, a 50-mL aliquot of the steel solution was transferred to a 250-mL beaker and heated on a hot plate. A hot 4% NaF solution (100 mL) was added to the beaker, and the mixture was stirred. The resulting white crystalline precipitate (5NaF⋅2FeF_3_) was removed by filtration through filter paper. The filtrate and the washings were transferred to a 250-mL volumetric flask and diluted to the mark with water. Aliquots of 1 mL were used to determine the cobalt content. 

### 3.3. CL-CPE–Spectrophotometric Optimization

The following solutions were successively added into a pre-weighed 50 mL conical tube: 1–12 mL of 10% TX-114, up to 1.4 mL of 4 × 10^−4^ mol L^−1^ Co(II), 3 mL of ammonium acetate buffer (with pH between 3.4 and 10.0), and 0.025–1.0 mL of 2 × 10^−3^ mol L^−1^ HTAR. The resulting solution was diluted to 50 mL with water and heated in a water bath for 5–40 min at ca. 50 °C. Then, the tube was placed in a refrigerator for 20–60 min (at −20 °C) to ensure completion of the precipitation process and easy removal of the supernatant by inverting the tube. After decantation, water was carefully added to the SRP to a total mass (SRP + H_2_O) of 5.00 g (an analytical balance was used for this operation). The mixture was then homogenized by gentle heating (for 1–2 min at 40–45 °C) and shaking. Finally, a portion of the resulting clear solution was poured into the cell, and the absorbance was measured against water or a simultaneously prepared blank.

### 3.4. Recommended Procedure for the Determination of Co

An aliquot of the analyzed solution (5.4–189 ng mL^−1^ Co) was placed in a pre-weighed 50-mL conical tube. Then, 8.2 mL of 10% Triton X-114 solution, 3 mL of the buffer with pH 7.8, and 0.25 mL of 2 × 10^−3^ mol L^−1^ HTAR solution were added. The tube was diluted to the mark (50 mL) with water and heated in a water bath (50 °C) for 20 min. After cooling in a refrigerator (at ca –20 °C) for 30 min, the supernatant was removed by inverting the tube. Water was carefully added to the SRP to a total mass (SRP + H_2_O) of 5.00 g, and the mixture was homogenized by gentle heating and shaking. A portion of the obtained clear solution was poured into the spectrophotometer cell, and absorbance was measured at 553 nm against a corresponding blank. The unknown cobalt concentration was calculated from a calibration plot.

## 4. Conclusions

A new extraction–chromogenic system for Co ions was studied. It is based on a novel commercially available hydrophobic azo dye, allowing the determination of trace cobalt. The proposed analytical procedure is simple, cheap, sensitive, and convenient. It is reliable and robust due to the wide optimal intervals of the examined variables. The use of organic solvents is not required, which characterizes it as green and environmentally friendly. Unlike other procedures requiring expensive and sophisticated instruments, the proposed analysis can be performed only with affordable and unpretentious equipment, such as a spectrophotometer, a water bath, and a refrigerator.

## Figures and Tables

**Table 1:** The CL-CPE–spectrophotometric optimization.

Parameter	Optimization Range	Optimal Value	Figure
Wavelength, nm	Visible range	553	1
pH	3.4–10.0	7.8	2
Concentration of HTAR, mol L^−1^	(0.1–4) × 10^−5^	1.0 × 10^−5^	3
Mass fraction of TX-114, %	0.2–2.4	1.64	4
Incubation time at 50 °C, min	5–40	20	5

**Table 2:** Effect of foreign ions on the determination of 4.7 μg Co(II).

Foreign Ion (FI) Added	Added Salt Formula	Amount of FI Added, mg	FI: Co Mass Ratio	Amount of Co found, μg	R, %
Al(III)	Al(NO_3_)_3_⋅9H_2_O	0.47	100	4.6	98.2
Ba(II)	Ba(NO_3_)_2_	47	10,000 *	4.9	105
Ca(II)	Ca(NO_3_)_2_	47	10,000 *	4.7	99.5
Cd(II)	CdCl_2_	0.094	20	4.9	105
Cr(III)	Cr_2_(SO_4_)_3_	0.094	20	4.5	95.0
Cr(VI)	K_2_CrO_4_	1.18 4.7	250 1000 *	4.7 4.5	101 95.8
Cu(II)	CuSO_4_⋅5H_2_O	0.0024	0.5	4.7	100
F^–^	NaF	47	10,000 *	4.9	105
Fe(III)	Fe_2_(SO_4_)_3_	0.024	5	4.8	103
HPO_4_^2^^–^	Na_2_HPO_4_⋅12H_2_O	9.4	2000	4.6	97.6
Hg(II)	Hg(NO_3_)_2_	0.235	50	4.6	97.1
Mg(II)	MgSO_4_⋅7H_2_O	47	10,000 *	4.8	101
Mn(II)	MnSO_4_⋅H_2_O	0.094	20	4.8	102
Mo(VI)	(NH_4_)_6_Mo_7_O_24_⋅4H_2_0	2.35	500 *	4.8	101
Na^+^	NaCl	47	10,000 *	4.7	99.0
Ni(II)	NiSO_4_⋅7H_2_O	0.0047	1	4.9	105
NO_3_^−^	NH_4_NO_3_	47	10,000 *	4.6	97.2
Re(VII)	NH_4_ReO_4_	4.7	1000 *	4.5	96.0
Pb(II)	Pb(NO_3_)_2_	0.047	10	4.5	95.8
V(V)	NH_4_VO_3_	0.0094	2	4.9	103
W(VI)	Na_2_WO_4_⋅2H_2_O	2.35	500 *	4.7	100
Zn(II)	ZnSO_4_⋅7H_2_O	0.0047	1	4.7	100

* Higher FI: Co mass ratios have not been investigated.

**Table 3:** Determination of cobalt in steel and dental alloy (*n* = 4).

#	Sample	Cobalt Content, %	Content of Other Elements (Manufacturer’s Data)	Cobalt Found *, %
1	Steel	4.71	17.7% W, 4.21% Cr, 1.58% V, 0.35% Mn, 0.081% C, 0.18% Si, and the balance Fe	4.77 ± 0.08
2	Dental alloy (Wirobond® C)	63.3	24.8% Cr, 5.3% W, 5.1% Mo, 1.0% Si, and Ce	62.8 ± 0.9

* Mean ± standard deviation (SD).

**Table 4:** Determination of cobalt in 1-mL Vitamin B_12_ (1000 μg) injection ampoules * (*n* = 6).

#	Present Method, μg Co per ampoule	RSD, %	ICP-MS **, μg Co per ampoule	RSD, %
Sample 1	44	4.4	45	5.5
Sample 2	44	5.9	45	5.5

* The calculated content of Co in 1000 μg of Vitamin B_12_ (C_63_H_88_CoN_14_O_14_P) is 43.5 μg. ** The analysis was performed in another laboratory.

**Table 5:** Addition–recovery of Co(II) from the rainwater and saline solution for intravenous infusion (*n* = 4).

Sample	Co(II) Concentration, ng mL^−1^		Recovery, %
	Added	Found *	
Rainwater	0	<LOQ	–
	20	21.5 ± 5.6	106
	40	41.1 ± 3.9	103
	60	59.3 ± 6.3	98.2
Saline solution for infusion	0	<LOQ	–
	20	20.8 ± 5.7	104
	40	40.9 ± 1.0	102
	60	59.6 ± 2.1	99.4

* Mean ± standard deviation (SD).

**Table 6:** Comparison with reported CPE–spectrophotometric procedures for the determination of cobalt(II).

Reagent(s)	Surfactant	SRP Diluting Agent	Sample	Wavelength, nm	Linear Range, ng mL^−1^	LOD, ng mL^−1^	Ref.
ACDA	TX-114	DMF	Water	452	20–200	7.5	[31]
APDC + C_16_MeImCl	TX-114	Ethanol	Water and alloy	598	150–2000	70	[41]
BTANP	TX-114	Methanol	Water	549	10–300	1.5	[35]
15-Crown-5	TX-114	Ethanol	Food	290	500–5000	400	[38]
MSE	TX-100	Ethanol	Water, biological samples	292	500–10,000	12	[40]
NaSCN	CTAB + SDS	Methanol	Tap and sea water	618	5890–35,400	6.18	[39]
N-BAEH	TX-100	Ethanol	–	294	500–10,000	12.7	[37]
PAN	TX-114	Ethanol	Water and urine	621	5–250	–	[32]
PAN	TX-114 + octanol	1 mol L^−1^ HNO_3_ in methanol	Water	450	2–300	0.6	[33]
Salen	TX-100	DMF	Wastewater	378	10–70	2.2	[34]
TPY	TX-114 + DOSS	Methanol	Tap and sea water	514	3140–18,960	4.54	[36]
HTAR	TX-114	Water	Dental alloy, steel, vitamin B_12_, rainwater, saline solution for infusion	553	5.4–189	1.64	This work

Abbreviations: ACDA, 2-amino-cyclopentene-1-dithiocarboxylic acid; APDC, ammonium pyrrolidine dithiocarbamate; BTANP, 2-(benzothiazolylazo)-4-nitrophenol; C_16_MeImCl, 1-hexadecyl-3-methylimidazolium chloride; CTAB, cetyltrimethylammonium bromide; DOSS, docusate sodium salt; MSE, methyl stearate ester; N-BAEH, N-benzoyl-L-arginine ethylester hydrochloride; PAN, 1-(2-pyridylazo)-2-naphthol; SDS, sodium dodecyl sulphate; TPY, 2,2’,6’,2”-terpyridine; TX-100, Triton X-100; TX-114, Triton X-114.

## Data Availability

Not applicable.
